# Open-Source and FAIR Research Software for Proteomics

**DOI:** 10.1021/acs.jproteome.4c01079

**Published:** 2025-04-23

**Authors:** Yasset Perez-Riverol, Wout Bittremieux, William S. Noble, Lennart Martens, Aivett Bilbao, Michael R. Lazear, Bjorn Grüning, Daniel S. Katz, Michael J. MacCoss, Chengxin Dai, Jimmy K. Eng, Robbin Bouwmeester, Michael R. Shortreed, Enrique Audain, Timo Sachsenberg, Jeroen Van Goey, Georg Wallmann, Bo Wen, Lukas Käll, William E. Fondrie

**Affiliations:** † European Molecular Biology Laboratory, European Bioinformatics Institute, Wellcome Genome Campus, Cambridge CB10 1SD, U.K.; ‡ Department of Computer Science, University of Antwerp, 2020 Antwerpen, Belgium; § Department of Genome Sciences, University of Washington, Seattle, Washington 98195, United States; ∥ VIB-UGent Center for Medical Biotechnology, VIB, Ghent 9052, Belgium; ⊥ Department of Biomolecular Medicine, Ghent University, Ghent 9052, Belgium; # Environmental Molecular Sciences Laboratory, Pacific Northwest National Laboratory, Richland, Washington 99352, United States; ⊗ US Department of Energy Agile BioFoundry, Emeryville, California 94608, United States; ¶ Belharra Therapeutics, 3985 Sorrento Valley Boulevard Suite C, San Diego, California 92121, United States; ○ Bioinformatics Group, Department of Computer Science, Albert-Ludwigs University Freiburg, Freiburg 79110, Germany; ● National Center for Supercomputing Applications & Siebel School of Computing and Data Science & School of Information Sciences, University of Illinois Urbana−Champaign, Urbana, Illinois 61801, United States; ◇ Department of Genome Sciences, University of Washington, 3720 15th St. NE, Seattle, Washington 98195, United States; ◆ State Key Laboratory of Proteomics, Beijing Proteome Research Center, National Center for Protein Sciences (Beijing), Beijing Institute of Life Omics, Beijing 102206, China; ■ Proteomics Resource, University of Washington, Seattle, Washington 98195, United States; □ Department of Chemistry, University of Wisconsin-Madison, Madison, Wisconsin 53706, United States; ▲ Institute of Medical Genetics, University Medicine Oldenburg, Carl von Ossietzky University, Oldenburg 26129, Germany; △ Department of Computer Science, Applied Bioinformatics, University of Tübingen, Tübingen 72076, Germany; ▼ InstaDeep London, London W2 1AY, U.K.; ▽ Proteomics and Signal Transduction, Max Planck Institute of Biochemistry, Martinsried 82152, Germany; α Science for Life Laboratory, School of Engineering Sciences in Chemistry, Biotechnology and Health, KTH Royal Institute of Technology, Stockholm 17165, Sweden; β Talus Bioscience, Seattle, Washington 98122, United States

**Keywords:** FAIR principles, open source, computational proteomics, best practices, data reuse, open data, mass spectrometry, proteomics

## Abstract

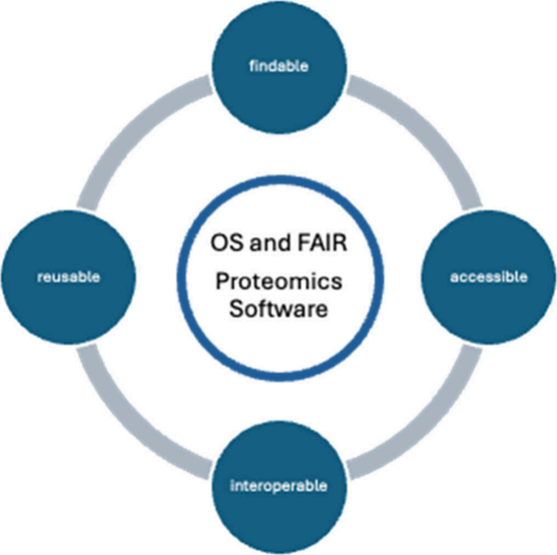

Scientific discovery relies on innovative software as much as experimental methods, especially in proteomics, where computational tools are essential for mass spectrometer setup, data analysis, and interpretation. Since the introduction of SEQUEST, proteomics software has grown into a complex ecosystem of algorithms, predictive models, and workflows, but the field faces challenges, including the increasing complexity of mass spectrometry data, limited reproducibility due to proprietary software, and difficulties integrating with other omics disciplines. Closed-source, platform-specific tools exacerbate these issues by restricting innovation, creating inefficiencies, and imposing hidden costs on the community. Open-source software (OSS), aligned with the FAIR Principles (Findable, Accessible, Interoperable, Reusable), offers a solution by promoting transparency, reproducibility, and community-driven development, which fosters collaboration and continuous improvement. In this manuscript, we explore the role of OSS in computational proteomics, its alignment with FAIR principles, and its potential to address challenges related to licensing, distribution, and standardization. Drawing on lessons from other omics fields, we present a vision for a future where OSS and FAIR principles underpin a transparent, accessible, and innovative proteomics community.

## 1 Introduction

Scientific discovery today is as much a product of innovative software as it is of groundbreaking experiments, and the right tools often mean the difference between success and stagnation. Indeed, the majority of scientists recognize scientific software as indispensable for their work and often impossible to conduct research without it.^1,2^ This reliance on software is equally crucial in proteomics, where researchers depend on a range of tools and algorithms for every step, from mass spectrometer configuration and data acquisition to the subsequent stages of processing, analysis, and interpretation.^3,4^

Since the original publication of the first mass spectrum database search tool, SEQUEST,^5^ proteomics software has evolved into a sophisticated ecosystem encompassing multiple stages of data processing, advanced predictive models, and robust computational frameworks. The publication of SEQUEST exemplifies a subsequent recurring pattern in MS-based proteomics, in which the development of software drives the adoption of novel experimental methodology. Numerous examples of open-source software tools have been developed and used by the proteomics community. These include tools like Percolator,^6^ which is used to improve peptide and protein identification using machine learning, MS2PIP^7^ and Prosit,^8^ which apply gradient boosting and deep learning, respectively, to predict fragment ion intensities, aiding in more accurate spectral matching, which can in turn be used by Percolator-based rescoring approaches like MS2Rescore,^9^ and Proteowizard,^10^ which provides shared libraries and tools for data access. Platforms like GalaxyP^11^ and quantms^12^ facilitate accessible, reproducible analyses through high-performance computing (HPC) and distributed workflows, supporting researchers in handling large data sets and complex analyses. Together, these advances underscore how proteomics software has transformed into a multidisciplinary field, involving a complex ecosystem of algorithms, models, and software tools that build upon sophisticated computational and algorithmic expertise.

Computational proteomics faces several key challenges common to other omics fields:

- The increasing complexity and size of data acquired by mass spectrometers,^13^ the complex sequence of steps, including spectral processing, statistical analysis, and biological interpretation, along with the need to manage algorithmic details and parameter settings,^14^ all contribute to making software development in proteomics a complex and demanding endeavor.
- Although most software tools are described in publications, the absence of open-source code, comprehensive documentation, and version control often impedes the reproducibility, reuse and interpretation of the results generated by these tools.^2^ This lack of transparency prevents researchers from extending existing algorithms and adapting software to keep pace with rapidly advancing instrumentation and acquisition methods.^15^ Transparency is especially important during the relatively frequent shifts in data processing paradigms, like the rapid adoption of data-independent acquisition over data-dependent acquisition.
- Ensuring reproducibility is a challenge due to the limited access to detailed algorithmic information, which hinders validation and extension of methods.^16^
- Custom licenses and restrictions on software distribution can further complicate the situation, making it difficult to share, modify, or redistribute software, and hindering the development of a collaborative and open-source ecosystem. Proteomics software is often distributed under restrictive licenses and tailored to specific platforms (e.g., operating systems, and computer architectures), limiting its use across diverse environments and services. Not surprisingly, such restrictive licenses hinder the field’s adaptability and impact the integration of proteomics with other omics disciplines.
- Complex workflows and high-throughput data analysis are growing in proteomics.^11,12,17^ This complexity requires managing dependencies, configurations, and environments consistently across diverse systems and architectures without human intervention. The community has tackled this challenge with continuous integration and deployment (CI/CD) pipelines as, for example, described in,^18^ but these pipelines rely upon the permission to freely redistribute software along the entire dependency chain. If one piece in this supply chain is not redistributable, then these exceptions must be handled, and automation is harder or impossible. A substantial additional burden is therefore created downstream of any non-OSS software package, which is a hidden cost on its own and one that affects the entire community. In sum, restricted distribution terms create an additional burden for the entire community downstream, which on its own is a hidden cost.
- The lack of standardization in proteomics software development, including inconsistent documentation,^19^ variable code quality, and limited community engagement, can hinder the adoption and use of software tools, leading to inefficiencies and redundancies in software development.^3^
- Closed-source, platform-specific software has caused lock-in effects, restricting users to specific tools and hindering innovation. Until recently, major instrument vendors lacked open-source, cross-platform libraries for data access, limiting data reuse and algorithm development.^20^ Thermo Fisher’s RawFileReader library marks important progress in this respect, enabling tools like ThermoRawFileParser^21^ and PRIDE Archive USI.^13,22^ This idea has been recently extended for Bruker timsTOF data with the timsrust library (https://github.com/MannLabs/timsrust/), which is open-source and already used by tools like the Sage search engine.^23^

A solution to these challenges is offered by open-source software (OSS) which is also aligned with the FAIR principles (Findable, Accessible, Interoperable, Reusable) that have initially been established for scientific data.^24^ The FAIR principles were expanded in 2022 to research software (FAIR4RS) to address the growing recognition of research software as a foundational research asset.^15,25^ Following FAIR4RS principles empowers proteomics with OSS tools that are not only accessible, but also foster community-driven development, rigorous validation, and transparent sharing of methodologies.^15,26^ Although OSS is not an explicit requirement for implementing FAIR principles, it facilitates the realization of these principles by making software more accessible, transparent, and reusable. OSS has demonstrated clear benefits in increasing the accessibility, usability, and visibility of scientific software. In particular, OSS makes reproducibility, traceability, and auditability possible. With code freely available for inspection, modification, and distribution, OSS encourages collaboration and creates avenues for continuous improvement—factors that are critical in fields as data-intensive as proteomics.

In this manuscript, we aim to explore the role of OSS in computational proteomics and its implications for the development of FAIR research software. We will discuss the benefits and challenges of OSS in proteomics, the role of OSS in the development of FAIR research software, and the importance of distribution, licensing, and citation of software in computational proteomics. We will also explore how other omics fields deal with OSS and FAIR software and how these experiences can inform the development of proteomics software. Our goal is to present a vision for a future where OSS and FAIR software are encouraged and supported in the proteomics community.

## 2 What Does It Mean for Software to Be “Open Source”?

### 2.1 Attributes of an Open-Source Project

Open-source software (OSS) is defined by its publicly accessible source code, allowing anyone to view, modify, and distribute it under an Open-Source Initiative-approved license. Merely making source code available is not enough; licenses that restrict use or modification to specific fields (e.g., noncommercial use) do not qualify as open source. Unlike closed-source, OSS guarantees transparency, fostering trust, collaboration, and scientific progress. Here, we outline the essential OSS criteria (https://opensource.org/osd) for the proteomics community, including users, developers, and reviewers:

- **Source Code Availability**: The source code—the instructions that define how software functions—is publicly accessible, allowing anyone to view, download, and examine the code’s details (https://opensource.org/osd).
- **OSI-Approved License**: The software must use an Open Source Initiative-approved license, which specifies rights to freely use, modify, and distribute the software, regardless of its application or environment (https://opensource.org/licenses).
- **Freedom to Modify and Distribute**: Open-source software, in contrast to source-available software, allows users not only to access the code but also to modify it and share these modifications, encouraging innovation and collaboration.
- **Transparency and Community Trust**: With open source, the code is transparent by design, allowing the community to understand, verify, and contribute to the project. This transparency fosters trust and credibility, which is essential in scientific fields.
- **Collaborative Development**: Open-source projects can be maintained by communities or dedicated teams, and they welcome contributions, such as bug reports, enhancements, and new features, from a diverse group of users and developers.
- **Long-term Sustainability**: Because the code is publicly accessible, open-source projects are less dependent on single organizations or developers for their maintenance and long-term survival, promoting continuity and stability even if the original contributors leave.
- **No Restriction to Specific User Groups**: Unlike “free-for-academic-use” licenses, which restrict usage to academic settings, open-source licenses do not impose limitations on the types of users or institutions that can access or use the software.
- **Not Necessarily Free of Cost**: Open-source software is “free” in terms of freedom, not necessarily in terms of price. Users might pay for support, hosting, or additional services, but they retain freedom in how they use and modify the software.

### 2.2 Misconceptions about Open Source

In proteomics and bioinformatics in general, multiple misconceptions exist about open/closed source software:

- Cost-free software is not always open-source: Many programs are freely available for noncommercial or academic use but do not meet open-source criteria. Similarly, ″free and open-source software″ (FOSS) refers to the freedom to run, modify, and share the software, not necessarily its financial cost. FOSS may involve expenses for services like support or hosting, but it ensures that users retain the freedom to use, adapt, and distribute the software as they wish (https://www.gnu.org/philosophy/free-sw.html).
- So-called ″academic licenses″ only refer to free-for-academic-use: The source code is not necessarily open, shareable, or modifiable. Even the term ″academic″ is not well-defined as it can refer to a wide range of institutions and organizations. To simplify this complexity, we can define OSS as any software that uses a license approved by the Open-Source Initiative (OSI, https://opensource.org/licenses).
- Accessible source code does not mean open-source: Open-source software does not only mean that the source code is available but that it is allowed to be freely modified and shared regardless of whether the users work in academic or commercial settings.
- Open-source software does not imply a lack of professional quality: Many open-source projects are maintained by dedicated teams with robust testing and good programming practices. In genomics, projects like samtools (https://github.com/samtools/samtools),^27^ an MIT-licensed (https://opensource.org/license/mit-0) project with over 80 contributors and 50,000+ citations, and the Genome Analysis Toolkit (GATK, https://github.com/broadinstitute/gatk),^28^ now open-source with over 100 maintainers and 26,000+ citations, exemplify this standard. In proteomics, Percolator (https://github.com/percolator/percolator)^6^ has over 2000 citations and 20 contributors, serving as a core tool for projects like MS2Rescore,^9^ OpenMS,^29^ MSBooster,^30^ DeepRescore,^31^ Crux,^32^ and even commercial tools like Mascot and Proteome Discoverer, and many others.^12,33,34^ Other successful open-source projects in proteomics, such as OpenMS,^29^ Skyline,^35^ Comet,^36^ PeptideShaker,^37^ ThermoRawFileParser,^21^ and ProteoWizard,^10^ demonstrate the benefits of transparency and collaboration. Despite these successes, academic open-source proteomics software is still perceived as lower quality. In 2018, Rob Smith highlighted the community’s concerns about academic proteomics and metabolomics software, including poor documentation, lack of transparency, and limited support.^38^ However, it should be noted that much of this feedback was directed at academic and free-for-academic-use software rather than exclusively open-source software.

### 2.3 Detrimental Practices in Using Public Repositories

In addition to the described misconceptions and complexity, many journals and some funding agencies mandate code availability as part of publishing, which has prompted multiple bad practices from software developers and bioinformaticians aiming to fulfill these requirements. Notable examples include:

- **Open-source Facade**: Researchers may upload closed-source software to platforms like GitHub, giving an impression of openness with features such as issue tracking, while the actual source code remains inaccessible. Although often well-intentioned, this practice can mislead scientists and, in our view, should be discouraged. In these instances, a clear statement in the repository should indicate to the users that the software is not open source.
- **Alterations Post-Publication**: Software is deposited in GitHub as open source during the submission of the manuscript, but after publication, software licenses in GitHub repositories are changed, or repositories are deleted or made private, all of which complicates efforts to ensure long-term accessibility.
- **License Misuse or Ambiguity**: Some repositories may use inappropriate or ambiguous licenses, causing confusion about the terms of use, distribution, and modification (more details discussed in the section Licenses in proteomics software).
- **Obscure Dependencies**: Software repositories may have dependencies that are not clearly documented, which may require closed-source or proprietary software. This can create barriers for other researchers attempting to run or build on software, as they may not have access to necessary components or may need to purchase expensive licenses. Clear documentation of all dependencies along with their licensing terms is essential to ensure transparency and reproducibility.

## 3 Licenses in Proteomics Software

We want to emphasize a fundamental aspect and challenge in proteomics software development: the choice of the licenses. Licenses serve as the foundation for defining key aspects of software, including commercialization, code reuse, distribution, and proper citation. It is therefore crucial to provide a license, and vital to choose a relevant one. As the gold standard for proteomics software development, we recommend using a standard OSS license like Apache 2.0, MIT, BSD, LGPL, and GPL; the full list of applicable licenses can be found at (https://opensource.org/licenses). These licenses are all well-known, are in use across many fields, and are well understood by the community. Additionally, they are compatible with the FAIR principles and the OSI guidelines.^15^ These established licenses moreover all have a clear definition of what is allowed and what is not, and how the software can be distributed, reused, and cited.

**Figure 1:**
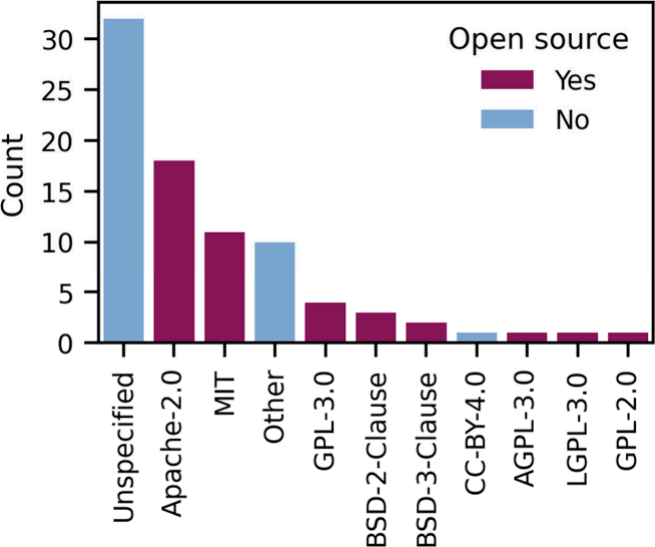
Software licenses in use in proteomics. Scientific papers published in the *Journal of Proteome Research* that include a GitHub URL in their abstract were automatically retrieved from PubMed and information on the software license of the corresponding GitHub repository was retrieved through the GitHub API. The code to generate these data is available at https://gist.github.com/bittremieux/70905e5d9dcc829ae49aab49e85954af.

Many proteomics code repositories do not have a software license specified (Figure 1). It is important to note that without a specified license the software is not open source. With an unspecified software license, the software and contributions are exclusively owned by the authors, and no one can use, copy, or distribute the contributions. The fact that so many proteomics tools have unspecified licenses underscores a misunderstanding of software licensing in the proteomics community.

In addition, as the field is evolving and software becomes more complex and has multiple components, different components could have different licenses. Consequently, dependencies between these components should be clearly stated. We recommend clearly stating the dependencies that a piece of software might have and the licenses of each of them. Full disclosure of such dependencies is necessary to ensure that the user is aware of this, such that the community, developers, and journal reviewers are able to understand this challenge.

## 4 Why Open-Source Software Is Essential for Scientific Research

### 4.1 Transparency Promotes Scientific Rigor

The scientific community increasingly recognizes that algorithms, while not software or tools themselves but rather the underlying steps and methodology, are becoming significant research outputs in their own right. Algorithms are no longer seen merely as tools but are valued as core research outputs, reflecting the critical steps and methodologies at the heart of scientific innovation. For example, the peptide spectrum scoring function HyperScore was originally implemented in the open-source search engine X!Tandem,^39^ later adopted by search engines including MSFragger,^40^ EncyclopeDIA,^41^ PepQuery^42^ and Sage.^23^ This shift highlights the importance of not only software as a means of implementation but also the reproducibility and reliability of the underlying computational methods that drive new discoveries. Both algorithms and their software implementations are now held to rigorous validation and reproducibility standards, similar to those for traditional experimental data and methodology.

Transparent computational methods open doors to innovation, enabling researchers to test hypotheses, refine methodologies, and build upon one another’s work with confidence. For instance, providing open-source implementations allows the scientific community to verify methods, adapt them to new challenges, and explore alternative approaches. Consider a proteomics experiment: without details on sample preparation or instrument settings or the raw data, the final results lack reproducibility. Similarly, open-source code ensures that computational methods can be accurately understood, replicated, and extended across laboratories worldwide. This transparency is particularly relevant for core proteomics workflows—as demonstrated by AlphaDIA^43^—where understanding the underlying algorithms of protein search engines directly impacts data interpretation and research outcomes.

When algorithms and models are shared as open-source software, they inherently uphold the FAIR principles applied to scientific data. This level of openness strengthens scientific rigor, enabling others to examine the code, replicate findings, and contribute improvements. A transparent approach to computational research, through openly available code, fosters a collaborative environment where the community can validate results and improve tools, ultimately building trust in computational methodologies and accelerating innovation.

Moreover, open-source implementations guard against unintended variation in outcomes caused by minor differences in coding practices, dependencies, or hardware environments. Even small programming choices can lead to significant changes in results. Open-source code mitigates these risks by making the entire process visible, allowing other scientists to understand the nuances and make informed adjustments. Transparency is key in computational research, not just for ensuring rigor but for building a reliable foundation that drives the entire field forward.

Finally, open-source code allows researchers to apply and compare different implementations, revealing assumptions and enhancing understanding. For instance, discrepancies between implementations of common tools, such as variations in BLOSUM matrices for sequence alignment,^44^ demonstrate how essential code transparency is for ensuring scientific consistency. Open-source practices thus empower researchers to expand on established methods with confidence, propelling science toward more robust, reproducible, and innovative outcomes.

### 4.2 Shared Knowledge Pushes the Field Forward

Open-source software fosters a collaborative ecosystem where researchers across institutions can freely contribute, refine, and extend tools, accelerating scientific progress. Unlike proprietary software that confines advances to specific laboratories or companies, OSS allows researchers to rapidly build on each other’s work without duplicating efforts, promoting efficient resource use and transforming individual achievements into collective gains. This is particularly vital in proteomics, where bioinformatics is integral to every workflow, and progress depends on the synergy between wet-lab experimentation and computational innovation. Extending and building on top of existing algorithms is crucial for scientific progress.

Proteomics has already greatly benefited from this open-source approach. Projects like ProteoWizard, with tools such as Skyline, msConvert,^45^ and SearchGUI,^46^ exemplify OSS’s impact. Skyline, for instance, supports over 20 external plugins available in its Tool Store, allowing users to perform specialized tasks far more efficiently than if they had to build solutions from scratch. Similarly, msConvert provides a standardized interface for mass spectrometry data, sparing developers the need to manage proprietary formats. SearchGUI, finally, provides a unified graphical user interface to 12 different search engines, in addition to ThermoRawFileParser and the above-mentioned msConvert. Together, such well-supported OSS projects create a foundational infrastructure that accelerates proteomics advancements.

The field of genomics offers a compelling example of how open-source initiatives can drive transformative progress. OSS such as reference-based aligners, e.g., BWA,^47^ variant-calling algorithms, e.g., GATK-HaplotypeCaller,^28^ and large-scale cloud-based genomic data analysis tools, e.g., Hail (https://hail.is), have revolutionized genomics research. Furthermore, these tools have been seamlessly integrated into broader computational frameworks like the nf-core/sarek,^48^ demonstrating how community-driven collaboration and standardization can amplify the impact of individual tools. This collaborative model underscores the potential for proteomics and other fields to follow a similar path, leveraging OSS to achieve greater integration, scalability, and innovation.

However, sustaining successful OSS projects in proteomics requires ongoing community engagement, which has often proven challenging. Despite their long history, projects like ProteoWizard and Skyline see few external contributions. Many researchers opt to develop independent tools rather than contribute enhancements within Skyline, missing opportunities for broader collaboration. Skyline’s external tools framework, which lowers technical barriers to contributions, has helped, but much of the development remains within the original laboratories. Community contributions in proteomics face barriers associated with multiple challenges. Developing software for proteomics demands specialized technical skills that many laboratories lack, especially when resources are focused on biological research rather than software engineering. The need for continuous updates to accommodate evolving data formats and instruments also requires substantial resources. Additionally, academic incentives often prioritize novel software creation over contributions to existing projects, further deterring collaborative development.

To create a more robust and impactful OSS ecosystem in proteomics, stronger incentives for community involvement and frameworks that support sustained collaboration are essential. With enhanced incentives, collaborative frameworks, and dedicated resources, the proteomics community can achieve a more sustainable, widely supported, and effective ecosystem of open-source tools. Apart from the engagement needed from the community to foster the development of open-source software, proteomics could create and sustain some of the core functionalities of the field in small libraries and tools that could be used by the entire community: for example tools like MS2Rescore for rescoring peptide identifications, pyOpenMS^49^ for Python-based proteomics functions, or spectrum_utils^50^ for spectral data manipulation.

### 4.3 The Community Can Contribute to Development

One of the greatest strengths of open-source software is that ″given enough eyeballs, all bugs are shallow″ (http://www.catb.org/~esr/writings/cathedral-bazaar/cathedral-bazaar/ar01s04.htmlhttp://www.catb.org/~esr/writings/cathedral-bazaar/cathedral-bazaar/ar01s04.html). Many of the critical pieces of software that underpin the modern technology stack are open source: Linux powers operating systems across the globe, Chromium serves as the foundation for multiple web browsers, PostgreSQL is a backbone of data storage, and Python and PyTorch have revolutionized machine learning and data science. Bringing this open-source ethos to proteomics holds the potential to accelerate advances in the field, creating tools that are not only robust but also accessible to a global community of researchers.

Bugs and mistakes are inevitable in complex software, but collaborative scrutiny allows them to be addressed more efficiently. In proteomics, as in other scientific fields, the diverse expertise of the community enhances both the quality and the utility of open-source tools. Users who encounter issues or limitations often provide feedback, suggest solutions, or even contribute code to address the challenges, fostering continuous improvement. In our own work, users have uncovered bugs that we subsequently corrected or asked questions about the underlying code which led to new features, fewer bugs, and more efficient algorithms. This open feedback loop is a defining feature of OSS, as community contributions not only help uncover and fix bugs but also actively shape the codebase and algorithms, fostering transparency and collaboration. Compared to proprietary software, OSS can often move faster and defray development costs by enabling users to build and contribute the features they need, rather than hoping that the maintainers of the software are willing or able to add the features themselves. This dynamic frees developers from the burden of predicting and implementing every possible use case and shifts some of the innovation to the broader community. For users, OSS reduces reliance on software maintainers, allowing research to advance even in the absence of formal support.

Without such transparency, computational research risks becoming a ″black box″ that stifles innovation rather than promoting it, hindering the growth of scientific knowledge. OSS can foster a culture of shared accountability, where code is not just released but continuously scrutinized and refined, driving the field forward in a collective effort toward scientific rigor. We have indeed observed this in some of our projects: at the time of writing, quantms and mokapot^51^ now have 12 and 13 contributors, respectively.

## 5 Open Source and ML/AI Models in Proteomics

Machine learning and deep learning are increasingly used in proteomics, with examples like the MS2 prediction models Prosit, pDeep,^52^ AlphaPeptDeep^53^ and MS2PIP, retention time prediction models DeepLC^54^ and AutoRT,^55^ and the *de novo* peptide sequencing models Casanovo^56^ and InstaNovo.^57^ Many deep learning-based proteomics tools enhance reproducibility by clearly reporting source code, training parameters, and other details. While closed-source tools have contributed to research, their models may carry biases that are difficult to detect and diagnose, and their potential utility can be hard to assess when code and models are not accessible. A more contentious issue arises when closed-source or commercial models are trained on publicly shared community data sets, often under open-source licenses.

Open-source software has proven its value by removing barriers to learning, sharing, and improving systems. For AI in proteomics, society needs similar freedoms: autonomy, transparency, ease of reuse, and collaborative improvement. The Open-Source Initiative’s Open-Source AI Definition (OSAID) outlines these freedoms:

- Use the system for any purpose.
- Study how the system works and inspect its components.
- Modify the system, including changing its output.
- Share the system, with or without modifications, for any purpose.

AI and machine learning are more than software: they encompass data, configurations, documentation, and artifacts like model weights and biases. ″Open source″ should apply to the entire system, including models, parameters, and structural elements. However, it is unclear what mechanisms or licenses ensure that these models, particularly their parameters, are freely available for use, research, modification, and sharing. We recommend clear assertions accompanying parameter distribution to ensure that they remain freely accessible.

## 6 Increasing Emphasis on Open Science and Open Source by Funding Agencies

As open science gains prominence, major funding agencies worldwide are implementing mandates to ensure that software developed with public funds is made openly accessible and reusable. Horizon Europe, the European Commission’s flagship research program, has set stringent requirements for open science, strongly recommending that research outputs, including software, are shared under open or free licenses aligned with FAIR principles (https://commission.europa.eu/about-european-commission/departments-and-executive-agencies/digital-services/open-source-software-strategy_en). Additionally, all Horizon Europe funded research is required to establish a data management plan (DMP), which is a structured document that outlines plans for open software and code sharing where possible, including tools needed for interoperability.

In the United States, agencies like the National Institutes of Health (NIH) and the National Science Foundation (NSF) strongly encourage, and in some cases require, software and code sharing through public repositories, aiming to maximize reproducibility and scientific transparency (https://datascience.nih.gov/tools-and-analytics/best-practices-for-sharing-research-software-faq). Similarly, the Wellcome Trust in the United Kingdom also recommends all research outputs, such as software integral to funded research, be available to ensure other research can verify it, build on it and use it to advance knowledge and make health improvements (https://wellcome.org/grant-funding/guidance/policies-grant-conditions/data-software-materials-management-and-sharing-policy). However, the same recommendations recognized that in some circumstances, controls and limits on sharing are necessary – for example, to protect the confidentiality and privacy of research participants, or to enable IP to be protected.

Many other funding agencies all over the world have similar open-source guidelines. This trend underscores a commitment from funders to foster collaborative scientific ecosystems, democratizing access to essential research tools and enhancing reproducibility across disciplines.

## 7 Challenges of Maintaining Open-Source Scientific Software

Open-source software in computational proteomics offers significant benefits but also poses challenges, particularly around sustainability. These challenges often deter long-term commitment, with some researchers transitioning to closed-source software after facing sustainability issues. Below, we outline key barriers to maintaining OSS and propose strategies—both practical and aspirational—to help advance OSS in the field.

### 7.1 Financial Sustainability

Maintaining an OSS project requires ongoing funding for updates, bug fixes, testing, and user support. However, funding agencies like the NIH often prioritize novelty over software maintenance, leaving many projects to become ″abandonware″ once the initial grant(s) end.

**Problem**: Without consistent funding, OSS projects in proteomics lose momentum after the initial development phase.

**Potential Solutions**:

- **Dedicated Maintenance Grants**: Funding agencies should offer grant mechanisms for software maintenance, such as the Chan Zuckerberg Initiative’s ″Essential Open-Source Software for Science″ grants. For example, the NIH previously supported software maintenance through an R01 mechanism, and today has a program to support sustainable OSS projects (https://grants.nih.gov/grants/guide/rfa-files/RFA-OD-24-010.html) directly.
- **Commercialization Models**: OSS projects could explore commercialization, potentially leading to academic spin-offs or new revenue streams (read the section about commercialization strategies).

### 7.2 Misaligned Incentive Structures in Academia

The academic incentive structure prioritizes publications and novelty, encouraging researchers to develop new software instead of maintaining existing tools. Contributions to OSS, especially those owned by others, are undervalued and rarely recognized in tenure or promotion evaluations.

**Problem:** The ″publish-or-perish″ culture discourages OSS maintenance, as it does not align with traditional academic metrics.

**Potential Solutions**:

- **Recognition for OSS Contributions**: Institutions and funding agencies should acknowledge OSS maintenance as valuable scholarly work, similar to publications, and include it in grant and tenure evaluations, as is, for instance, the case in the European Commission’s ERC program CV template.
- **Community-driven Publications**: Journals should accept papers on software updates, offering academic recognition for maintenance work, as seen in the Journal of Proteome Research’s Software Tools and Resources issue.

### 7.3 The Challenge of Consistent Maintainers

In academic settings, many OSS projects are led by students, postdocs, or temporary researchers who eventually leave for other opportunities, often in unrelated fields. This results in a lack of long-term maintainers, leading to project stagnation or abandonment.

**Problem:** The reliance on transient academic positions means OSS projects are vulnerable to disruptions as contributors move on.

**Potential Solutions**:

- **Governance Models**: Establishing community-driven governance structures, such as steering committees or core maintainer teams, can provide continuity even as individual contributors leave. Notably, this kind of governance is likely only feasible for larger, well-established open-source projects.
- **Transition Plans**: Projects should develop clear transition plans, ensuring that new maintainers can seamlessly take over. This could involve thorough documentation, onboarding guidelines, and mentoring new contributors.

Addressing these challenges requires a multipronged approach, combining changes in funding structures, academic incentives, and community engagement. The scientific community, funding agencies, companies, and academic institutions must collaborate to ensure that OSS can continue to thrive. By addressing these challenges head-on, we can build a more sustainable and collaborative ecosystem for open-source scientific software, ultimately driving innovation and reproducibility in proteomics research.

## 8 How to Start a Gold-Standard OSS Project in Proteomics

The following steps in Box 1 provide a guideline that can foster a successful open-source project that grows in adoption, value, and contributions over time.

Box 1 **How to get started with OSS.**

1- **Define clear goals and scope**: Start by defining the specific problem or gap your software aims to solve. Ensure it addresses an unmet need or provides a significant improvement over existing solutions. Before starting an independent OSS project, consider contributing to an existing OSS project by evaluating if your use case could take advantage of existing frameworks. For example, adding a new feature within Skyline or OpenMS would not require using your resources for implementing a raw data reading component and a user interface.
2- **Choose an open-source license**: Choose an OSI approved license that aligns with the project’s intended use and desired level of openness. For projects that may later require commercialization or enterprise use, dual licensing (e.g., open source with an option for commercial licensing) can be considered to support sustainability.
3- **Plan for sustainability**: Research potential funding sources, such as grants, academic support, or partnerships. Decide if the project will rely on donations, grants, or if it might later incorporate paid services. If applicable, consider models like SaaS, support-based revenue, or feature-based licensing that could sustain the project without sacrificing its open-source nature.
4- **Set up a well-structured repository:** Use a version-control platform like GitHub or GitLab for easy access, community contributions, and versioning. Use clear folder structures, name conventions, and modular code design to enhance usability and maintainability. Provide a clear guide on how others can contribute to the project, including coding standards, pull request policies, and a Code of Conduct to foster a positive collaborative environment.
5- **Incorporate early user feedback**: Develop a prototype and engage a select group of users as beta testers than can try the software and provide feedback to ensure its usefulness and effectiveness.
6- **Implement rigorous testing and quality control**: Use CI/CD practices like GitHub Actions to automate testing and improve code quality. Create robust tests to ensure functionality and compatibility and regularly review code with input from experienced contributors or collaborators.
7- Develop thorough documentation:
  a. **User documentation**: Provide tutorials, installation guides, and usage examples that lower barriers to entry for new users.
  b. **Developer documentation**: Include technical details that make it easier for new developers to understand the codebase, contribute, and debug.
  c. **Version control and changelog**: Maintain a detailed changelog for tracking updates, and consider using semantic versioning for releases to help users track changes and updates.
8- **Build a community**: Create forums, mailing lists, or a Slack channel to facilitate communication and support for users and contributors. Promote the project within academic and industry circles, social media, or conferences. Encourage diverse participation, whether from seasoned developers, scientists, or students, by being open to questions, feedback, and contributions of varying levels.
9- **Ensure long-term maintenance and evolution**: Provide a roadmap to outline planned features and long-term goals, keeping contributors aligned and users confident. Build an engaged community by recognizing contributors, hosting events, and welcoming new ideas. Adopt a governance model, such as a core maintainer group, to ensure the project’s mission endures despite contributor changes.
10- **Monitor and measure success**: Track metrics like repository stars, downloads, citations, or code contributions to gauge adoption and impact. Regularly collect user feedback and address concerns or feature requests to ensure the project stays relevant and useful to its audience.
11- **Stable DOIs:** To prevent issues with license or code changes after publication, OSS projects should use archival platforms like Zenodo, Figshare, or Software Heritage, which offer DOIs for long-term citation and access. These platforms integrate with GitHub for automated, enduring accessibility.

## 9 Creation of Sustainable, Open-Source Software in an Academic Setting

A primary purpose of the academic laboratory is the training of graduate and postdoctoral students. These positions are by their nature, of limited duration. The creation and development of software tools can be an ideal mechanism for creating a deep understanding of the concepts and best practices of proteomics. However, tools created during training can languish following the graduation and departure of the student unless there is a considered and established plan for sustainability in place. We have established and maintained a procedure for sustainable software using the following established practices (Box 2).

Box 2 **One working approach to sustainable software development in an academic setting**

1- All students create code in the same language.
2- The language used by the lab should operate across major platforms (Windows, Linux, and MacOS).
3- All new code must make maximal use of existing code for efficiency.
4- Whenever possible, new tools developed by students or staff are integrated into the core codebase rather than downstream applications, ensuring broader usability across multiple projects. All contributors must agree to make their work available and receive proper credit for their contributions.
5- All adaptations of existing code or newly created code must be covered by unit tests that become a permanent part of the code base.
6- All new code must be reviewed and approved by an additional member of the team through code reviews.
7- All code must pass nightly build tests before public release.
8- New applications should be extensions of existing applications whenever possible.

The rationale for these rules is as follows. Choosing a single language for the laboratory means that all students will be well-versed and deeply knowledgeable in that language. This enables an easy understanding of existing code and the ability to understand the code written by other contributors. Reusing an established codebase eventually results in robust, reliable, and bug-free operation. Moreover, all contributors become extremely conversant with the individual capabilities and their straightforward and facile integration into new tools. Student contributions are guided by the group’s consensus, being incorporated into our codebase where they make the most sense and with an eye toward their future use. The requirement for unit test coverage means that new code functions as expected and maintains the functionality of existing functions. The requirement for three reviews means that many other lab members well understand all code created in the lab. Therefore, when a student leaves the lab, there are many individuals still around who understand all that student’s code and can maintain it moving forward. The requirement that students extend projects with new functionality rather than create stand-alone software provides an avenue to reuse established code with proven reliability, limiting potential bugs only to the new portions of code. The effect of some code changes cannot be predicted. Therefore, the use of nightly build tests, where many code operations are evaluated with large data sets enable the team to find unexpected changes to the results or operation time. A key ancillary benefit of maximizing code reuse and minimizing new monolithic applications is the great reduction in the amount of code that needs maintenance over the long-term. Code maintenance can require a significant investment of capital and human resources. Therefore, for the academic lab, a concerted effort to reduce the need for both of those precious resources is vital.

## 10 Interoperability in Proteomics Software

An important part of FAIR principles is interoperability, which refers to the ability of data, metadata, and algorithms/modules to work together across different systems, applications, and disciplines. The proteomics community, led by multiple OS projects and developers, has developed and championed multiple formats, standards, and libraries over the years to enable the exchange of proteomics data. Most of these efforts have been triggered and coordinated under the HUPO Proteomics Standards Initiative (HUPO-PSI).^58^ The HUPO-PSI has developed multiple file formats including mzML,^59^ mzIdentML,^60^ mzTab^61^ and SDRF-Proteomics^62^ that enable the exchange of data in public repositories but also the development of new OS components, tools for visualization and analysis based on standardized formats.^63^

Interoperability in proteomics software development goes beyond standardized file formats—it also involves how data is represented. Standardized representations, such as ProForma^64^ to represent peptides and proteoforms; and Universal Spectrum Identifier (USI)^22^ a standardized identifier used to uniquely reference a mass spectrum across different repositories and data sets, play a crucial role in ensuring that peptides, spectra, and other proteomics data are consistently interpreted across different tools and databases. Multiple tools and OS components are using these standards to exchange peptides and spectra, including spectrum viewers and annotation tools.^13,42,50,65^

Adoption, contributions and extensions of standards file formats across open-source, close-source, and proprietary software vary depending on the standard and its scope. mzML (https://www.psidev.info/mzml), for example, is widely supported by a variety of tools of different nature, including FragPipe/MSFragger,^40^ DIA-NN,^66^ Mascot, PEAKS,^67^ OpenMS, quantms, Galaxy-P. However, it could be seen that OS tools, libraries, and components considered from the very beginning to support standard file formats compared with other closed-source tools; in the case of mzML this could be observed by the amount of OS tools that support its development but also use it as a first-class format for spectrum file, for example, OpenMS,^29^ quantms,^12^ Crux,^32^ comet,^36,68^ MSGF+,^33,69^ MyriMatch,^70^ Sage,^23^ PepQuery.^42^

By addressing data integration challenges, the OSS community plays a vital role in developing proteomics data formats, such as mzML, mzTab, ProForma, and USI. Their collaborative initiatives, such as HUPO-PSI^58^ and EuBIC,^71^ have pioneered the creation of standard OS libraries to write and validate them, making proteomics data more accessible and reusable. OSS-driven standards highlight the value of community in ensuring that data is FAIR (Findable, Accessible, Interoperable, and Reusable), promoting long-term sustainability and interoperability across diverse analytical workflows. As the field evolves, the continued engagement of the OSS community will be crucial in maintaining and improving these standards, ensuring that proteomics data remains transparent, reproducible, and integrable to future research and technological advancements.

## 11 Strategies to Commercialize OSS

Open-source software (OSS) is not free from costs; maintaining, running, and developing it requires resources. To ensure long-term sustainability, several commercialization strategies have been developed, balancing openness with financial viability, in a manner suiting the needs of the owner. Here, we consider ″commercialization″ as any means to monetize OSS, whether it remains in an academic setting, is adopted by a company, or spun out into a startup. We argue that healthy OSS projects must be financially supported by methods such as charitable means, grants, or commercialization, for the development of the project to be sustainable. We discuss a few commercialization models that have become popular with OSS, which try to strike a balance between supporting openness and supporting future development. It is worth noting that these strategies are not necessarily mutually exclusive.

- **Dual licensing**: A popular commercialization option for OSS has been to offer the software under both a strong copyleft license (like GPL or AGPL) and a more permissive commercial license. The code itself is typically the same for both license types. The difference lies in how the code can be used, modified, and redistributed depending on the license under which it is acquired. Projects using this strategy are often available under a strong copyleft license (GPL, AGPL, etc.) with no financial cost. However, the copyleft nature of these licenses requires any derivative works to be published under a compatible open-source license, which is often undesirable for corporate users. Thus, projects also offer more permissive commercial licenses to paying customers, allowing them to use the OSS project within proprietary code. Although this approach may seem prone to abuse (e.g., improper use of GPL code), our experience has been that companies tend to be risk-averse and prefer purchasing proper licenses to avoid violating a copyleft license. A successful example of this strategy from outside of proteomics has been RStudio by Posit. RStudio is currently available under an open-source AGPLv3 license, or a commercial license when AGPLv3 is incompatible. Notably, developers should make sure to include a ″contributor license agreement″ as part of their requirements for new contributors to ensure their contributions can be distributed under both licenses.
- **Support or services**: Some OSS projects commercialize by offering support services or new feature development at a cost. Often users, particularly from corporate entities, are willing to pay for specialized training and ongoing support for their use of OSS projects. In special instances, it may even be the case that outside entities can pay for the prioritization of specific features. For example, the major mass spectrometry instrument vendors have been providing financial support to both the Skyline and ProteoWizard projects to ensure features, support, and documentation are provided for their customers. This road must be trod carefully though; while there is a benefit to allowing sponsored features, and they do benefit everyone once implemented; such a model risks losing control over the direction of an OSS project. Features added to Skyline from a vendor are made available to all vendors if they have compatible instrumentation. Red Hat is the most prominent example of a company using this strategy to commercialize its enterprise Linux offering.
- **Software as a service (SaaS)**: The SaaS commercialization model has become increasingly popular in recent times. When using a SaaS model, the OSS project remains open source, but commercialization occurs by building a platform around it. The platform then allows users to more easily use the OSS project. This model often includes a managed hardware or cloud infrastructure component, where users pay to interact with a web application to use the OSS tool, reducing the barrier to entry. In the bioinformatics space, NextFlow^72^ is an open-source bioinformatics workflow engine that has been commercialized by Seqera Laboratories using the SaaS model. Their current Seqera Platform product provides an interface to launch, observe, and explore workflow executions with NextFlow, in addition to other features.
- **Open-core**: The open-core commercialization model provides access to new features only to paying customers. Rather than essential functionality, this refers to optional features such as a nicer user interface or early access to new features. Some variants of this model use a time delay for new features, where paying users have access to new features sooner than those using the fully OSS version. Practically, the implementation of this strategy often involves the creation of a private, upstream fork of the OSS code repository. New features are then added to the private fork and synced to the OSS version at a later date. Such a strategy can also be used by academic laboratories looking to protect new features while preparing for publication and until a manuscript is accepted. Although we advocate for developing those features in the open, we recognize that there are instances where this is not practical. For example, when a junior researcher is publishing a novel algorithm, they may want to avoid the risk of having their work pre-empted by others. Similarly, collaborators may request that the software be kept private to prevent other researchers from using it and publishing their findings first. While we believe that these situations are rare in the proteomics community, they could lead to the original researchers losing recognition and credit for their work. The open-core model is quite common, and in proteomics, it is used for ScaffoldDIA from Proteome Software: the open-source core of ScaffoldDIA is EncyclopeDIA.

## 12 Concluding Remarks

As proteomics increasingly depends on computational tools, adopting open-source and FAIR principles is crucial for ensuring transparency, reproducibility, and accessibility. We urge researchers, funding agencies, institutions, and companies to prioritize open-source practices, particularly for publicly funded work, to foster a truly collaborative scientific ecosystem. By collectively advancing open-source software, the scientific community can build an inclusive, rigorous foundation that fosters innovation and extends the benefits of research to scientists and the public alike.

Moving forward, we as a community should explore mechanisms to make OSS sustainable, for example, by creating a foundation for proteomics software to support the maintenance of OSS in our field. Emphasizing scalable, user-friendly software with complex features hidden behind intuitive interfaces will help ensure widespread adoption and success.^73^ This approach can also counteract negative perceptions of the quality of academic or OSS in mass spectrometry.^38^ Additionally, we expect that AI-assisted software development will enhance the quality of proteomics OSS by automating error detection, optimizing code performance, and enhancing feature integration—ultimately boosting reliability and user satisfaction. Regardless, let us unite in our commitment to open science and pursue a shared, sustainable future in our exploration of the proteome.
